# Bioformation of Volatile and Nonvolatile Metabolites by *Saccharomycopsis fibuligera* KJJ81 Cultivated under Different Conditions—Carbon Sources and Cultivation Times

**DOI:** 10.3390/molecules23112762

**Published:** 2018-10-25

**Authors:** Sang Mi Lee, Ji Hye Jung, Jeong-Ah Seo, Young-Suk Kim

**Affiliations:** 1 Department of Food Science and Engineering, EwhaWomans University, Seoul 120-750, Korea; smlee78@ewha.ac.kr (S.M.L.); 923jjh@gmail.com (J.H.J.); 2 School of Systems Biomedical Science, Soongsil University, Seoul 06978, Korea; sja815@ssu.ac.kr

**Keywords:** *Saccharomycopsis fibuligera* KJJ81, *nuruk*, volatile metabolites, nonvolatile metabolits

## Abstract

*Saccharomycopsis fibuligera* KJJ81 isolated from *nuruk* is an amylolytic yeast that is widely used as a microbial starter in various fermented foods. Volatile and nonvolatile metabolites of *S. fibuligera* KJJ81 were investigated according to different carbon sources and cultivation times using a nontargeted metabolomic approach. Partial-least-squares discriminant analysis was applied to determine the major metabolites, which were found to be closely related to the clustering and discrimination of *S. fibuligera* KJJ81 samples. Some volatile metabolites derived from phenylalanine, such as 2-phenylethanol, 2-phenylethyl acetate, and ethyl phenylacetate, were predominantly found in cultivation medium containing glucose (YPD medium). In addition, the level of 2-phenylethanol increased continuously with the cultivation time. In terms of nonvolatile metabolites, carbohydrates (mannose, arabitol, and mannitol), fatty acids (palmitic acid and stearic acid), organic acids (oxalic acid and succinic acid), and amino acids (isoleucine, serine, alanine, glutamic acid, glycine, proline, phenylalanine, and threonine) were the main contributors to *S. fibuligera* KJJ81 samples cultivated in YPD medium according to cultivation time. These results show that the formation of volatile and nonvolatile metabolites of *S. fibuligera* KJJ81 can be significantly affected by both the carbon sources and the cultivation time.

## 1. Introduction

*Makgeolli* is a traditional alcoholic beverage made in Korea from the fermentation of rice after the addition of *koji* or *nuruk* [1]. *Koji* is a kind of microbial starter that includes a single culturing microorganism, whereas *nuruk* is a traditional microbial mixed-strain starter that is primarily cultivated on rice, wheat, barley, and rye [2]. This means that *nuruk* includes diverse air-borne naturally occurring microorganisms such as filamentous fungi, yeasts, and bacteria [2,3]. *Makgeolli* is generally manufactured by fermentation involving saccharification and alcoholic fermentation. Enzymes such as amylase and glucoamylase can be used for saccharification—along with decomposition of protein by protease—in rice [4]. During alcoholic fermentation, yeast can produce ethanol, carbon dioxide, and diverse compounds including fusel alcohols and esters that can affect the quality of *makgeolli* [4,5]. In addition, the taste and flavor characteristics of *makgeolli* are due to a complex mixture of nonvolatile and volatile metabolites whose formation can be affected by the microbial activities during fermentation, mainly due to differences in enzyme activities [6,7]. Therefore, microorganisms in *nuruk* or *koji* that can act as enzyme sources are critical for the fermentation of *makgeolli*.

*Saccharomycopsis fibuligera* is one species of the teleomorphic ascomycetous genera [8,9]. The food-borne microorganism is a representative producer of amylolytic enzymes among ascomycetous yeasts, and is widely used as a microbial starter in various fermented foods [1,9,10,11]. In particular, *S. fibuligera* has the ability to accumulate trehalose from soluble starch and produce amylase, acid protease, and β-glucosidase [9,12]. *Saccharomyces* species are the predominant yeast species in *nuruk*, and their effects on the quality of *nuruk* and *makgeolli* have been studied [7,9]. However, although several studies have investigated the metabolites of *Saccharomyces cerevisiae* in *nuruk* and *makgeolli*, no previous study has investigated the formation of metabolites of *S. fibuligera*.

Microbial metabolite profiles and also the effects of cultivation conditions on the formation of metabolites produced by microorganisms have been analyzed using various high-throughput analytical methods such as gas chromatography (GC), gas chromatography–mass spectrometry (GC-MS), high-performance liquid chromatography, and capillary electrophoresis [13,14,15]. Vilanova et al. determined that the effect of ammonium supplementation of a synthetic medium on the production of volatile and nonvolatile compounds by strains of *Saccharomyces cerevisiae* during wine fermentation [16]. It was reported that branched-chain fatty acids and their esters were associated with low nitrogen concentrations, whereas fatty esters comprising chains of medium length and acetic acid were related to high nitrogen concentrations. In addition, the formation of diverse metabolites can be affected by cultivation media containing different carbon sources as well as by the type of microorganisms [17,18].

The objective of the present study was to determine the volatile and nonvolatile metabolites of *S. fibuligera* KJJ81 that can be isolated from *nuruk* using a nontargeted metabolomic approach. Also, differences of the volatile and nonvolatile metabolites of *S. fibuligera* KJJ81 were compared according to the carbon sources and cultivation times. The results obtained will promote the understanding and utilization of *S. fibuligera* KJJ81, which might be related to the organoleptic properties of fermented products.

## 2. Results and Discussion

### 2.1. Effects of Carbon Sources on the Formation of Volatile Metabolites by S. fibuligera KJJ81

Carbohydrates are important sources of both carbon and energy for cultured microorganisms [18]. In order to identify a suitable carbon source for the formation of volatile metabolites by the cultivation of *S. fibuligera* KJJ81, the present study obtained growth curves of *S. fibuligera* KJJ81 cultivated in media containing different carbohydrates such as glucose, fructose, galactose, xylose, and maltose. 

**Figure 1:**
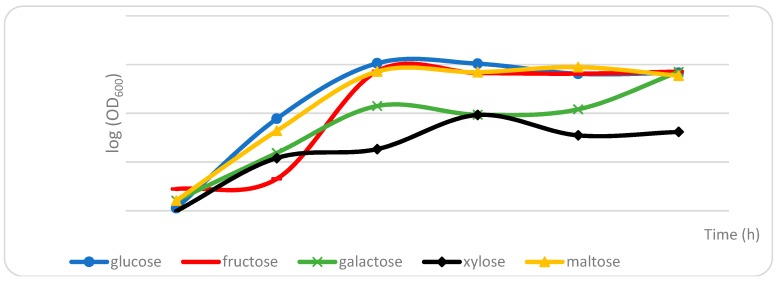
Growth curves of *S. fibuligera* KJJ81 cultivated in different types of media containing different carbon sources such as glucose, fructose, galactose, xylose, and maltose.

**Figure 2:**
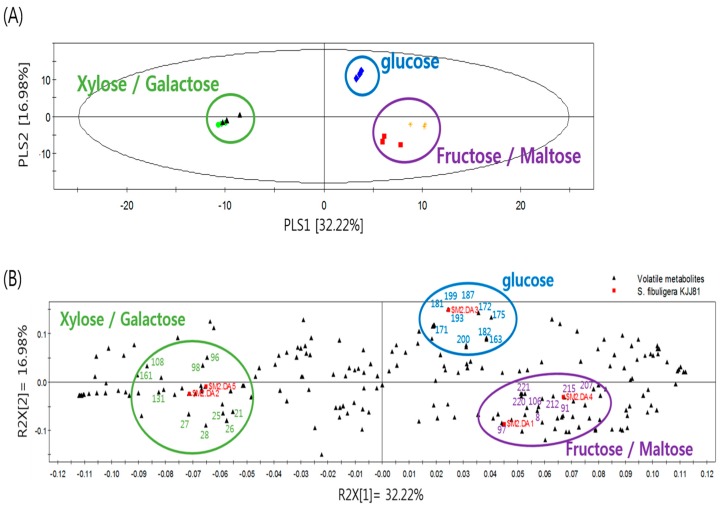
PLS-DA score plot (**A**) and loading plot (**B**) obtained from GC-MS data for volatile metabolites in the extracts of *S. fibuligera* KJJ81 cultivated in media containing different carbohydrates.

The growth of *S. fibuligera* KJJ81 reached a stationary phase after 18 h (Figure 1), which is consistent with the time point that is known to be optimal for analyzing secondary metabolites [19]. A comprehensive nontargeted analysis of all the volatile metabolites in the extracts of *S. fibuligera* KJJ81 cultivated in media containing different carbohydrates was performed using GC-MS equipped with stir bar sorptive extraction (SBSE). GC-MS data were processed using software of the Automated Mass Spectral Deconvolution and Identification System (AMDIS), which provides the abilities to perform deconvolution, adjacent peak deconvolution, and retention index comparison. Figure 2 presents a score plot from the partial-least-squares discriminant analysis (PLS-DA) of GC-MS data sets that were obtained from *S. fibuligera* KJJ81 cultivated in different media for 18 h. Two PLS components explained 32.22% (PLS 1) and 16.98% (PLS 2) of the total variance. PLS-DA cross-validation was performed to ensure model reliability. The parameters of the cross-validation modeling for the third PLS component were R2X = 0.592, R2Y = 0.738, and Q2Y = 0.455. Permutation testing (100 iterations) was also performed in order to further validate the model, after which R2 = 0.315 and Q2 = −0.364. As shown in Figure 2, *S. fibuligera* KJJ81 samples cultivated in media containing glucose, fructose, or maltose could be distinguished from those cultivated in media containing xylose or galactose along the PLS 1 dimension. Differences of volatile metabolites in the three separated groups are displayed in the loading plot in Figure 2. The major volatile metabolites were identified according to VIP values (with a criterion of VIP > 0.7) (Table 1).

Some volatile metabolites such as 1-octanol, 1,3-bis(2-methyl-2-propanyl)benzene, (2*E*,6*E*)-3,7,11-trimethyl-2,6,10-dodecatrien-1-yl acetate, 4-(2-methyl-2-propanyl)cyclohexyl acetate, and 5-[(2*E*)-2-octen-1-yl]dihydro-2(3*H*)-furanone were detected in *S. fibuligera* KJJ81 samples cultivated in media containing xylose and galactose. In addition, 4 alcohols (1-octen-3-ol, 2-furylmethanol, 3-methyl-1-butanol, and phenol), 1 ester (3-methylbutyl acetate), 1 furan [5-hexyldihydro-2(3*H*)-furanone], and 1 sulfur-containing compound were identified in samples cultivated in media containing fructose and maltose.

3-Methyl-1-butanol (isoamyl alcohol), which is well known to be one of the major odorants in whiskey, can be derived from glucose metabolism by yeast fermentation [20]. 3-Methylbutyl acetate (isoamyl acetate) with sweet and fruity odor notes can be produced via the metabolic pathway as 3-methyl-1-butanol [21]. In particular, relatively high contents of 3-methyl-1-butanol and 3-methylbutyl acetate were produced in the fructose and maltose groups (data not shown). 3-Methyl-1-butanol and 3-methylbutyl acetate were derived from carbohydrates and amino acids (valine and leucine) and produced via pyruvate, α-ketoisovalerate, and α-ketoisocaproate [20,22]. Many enzymes were involved in the degradation process to pyruvate, including hexokinase, phosphoglucose isomerase, and pyruvate kinase [21]. Moreover, diverse enzymes—including alcohol acetyltransferase, acetate kinase, phosphotransacetylase, and coenzymes comprising acetyl CoA—were required to produce 3-methylbutyl acetate [21]. It was found that 3-methylbutyl acetate in wine-making yeasts is synthesized only in the presence of acetyl-CoA [23].

1-Octen-3-ol, which has a mushroom-like odor note, is formed mainly from linoleic acid and linolenic acid via a pathway that converts linoleic acid 10-hydroperoxide into 1-octen-3-ol [24]. The neutral lipids are hydrolyzed into unsaturated free fatty acids such as linoleic acid and linolenic acid by acyl hydrolases. Polyunsaturated fatty acids are subsequently oxygenated with hydrogen peroxide catalyzed by lipoxygenase, and finally cleaving enzymes (e.g., hydroperoxide lyase, oxidoreductases, and isomerases) transform hydroperoxide (linoleic acid 10-hydroperoxide) into volatile alcohols and aldehydes [24].

5-Hexyldihydro-2(3*H*)-furanone (γ-decalactone) was detected as a major volatile metabolite of samples cultivated in media containing fructose and maltose. 5-Hexyldihydro-2(3*H*)-furanone is responsible for a peach-like odor note, and it is normally present in various fruits and fermented products [25]. 5-Hexyldihydro-2(3*H*)-furanone is an intermediate of ricinoleic acid catabolism (peroxisomal β-oxidation of ricinoleic acid) [25]. Romero-Guido et al. reported that the formation pathway of 5-hexyldihydro-2(3*H*)-furanone involves oleic acid being converted into ricinoleic acid via hydroxylation, and then changed to 4-hydroxydecanoic acid by β-oxidation and isomerization, with 4-hydroxydecanoic acid finally being transformed into 5-hexyldihydro-2(3*H*)-furanone via lactonization [26].

The major volatile metabolites in Table 1 shows that two alcohols [2-phenylethanol and (2*E*)-3,7-dimethyl-2,6-octadien-1-ol], three aldehydes (3-dodecenal, octanal and 2-octenal), three esters (2-phenylethyl acetate, ethyl phenylacetate and ethyl acetate), and one lactone (6-heptyltetrahydro-2*H*-pyran-2-one) were strongly associated with *S. fibuligera* KJJ81 samples cultivated in medium containing glucose. In particular, volatile metabolites with phenyl group such as 2-phenylethanol, 2-phenylethyl acetate, and ethyl phenylacetate were detected as major volatile metabolites in media containing glucose. 2-Phenylethanol, which is derived from the degradation of phenylalanine, occurs naturally in beverages such as beer, wine, and whiskey with a rose-like odor note [2], and is also detected as an odor-active compound in makgeolli [5]. *Saccharomyces*, *Kluyveromyces*, *Pichia*, and *Aspergillus* species can produce 2-phenylethanol [27]. Viana et al. reported that 2-phenylethanol can be converted into 2-phenylethyl acetate by alcohol acetyltransferase, whereas esterase catalyzes the hydrolysis of 2-phenylethyl acetate into 2-phenylethanol and acetic acid [28]. 2-Phenylethyl acetate, which is well known to be one of the major odorants in wine, has honey-like, fruity and flowery odor notes [29]. Chen and Xu reported that ethyl esters, which have fruity and flowery odor notes, constituted the largest group of volatile compounds in Chinese rice wine [29]. Saerens et al. found that the transfer of ethyl esters formed intracellularly by yeast cells into the fermenting medium decreased markedly with increasing chain length [30].

The above results indicate that various volatile metabolites are detected in *S. fibuligera* KJJ81 cultivated from media containing different carbon sources. It is assumed that different carbon sources might exert different effects of catabolic repression on the cellular secondary metabolism. In particular, volatile metabolites with phenyl groups and derived from phenylalanine, such as 2-phenylethanol, 2-phenylethyl acetate and ethyl phenylacetate, were predominantly found in glucose cultivation medium (YPD medium). Most of these volatile metabolites have characteristic fruity and flowery odor notes, which affect the quality of *makgeolli* after fermentation [7]. The present study focused on the effects of carbon sources with the aim of improving the formation of those volatile metabolites with fruity and flowery odor notes by the cultivation of *S. fibuligera* KJJ81. Accordingly, the volatile and nonvolatile metabolites of *S. fibuligera* KJJ81 in YPD medium were further investigated according to the cultivation time.

### 2.2. Cultivation-Time-Dependent Volatile Metabolites of S. fibuligera KJJ81 in YPD Medium

**Figure 3:**
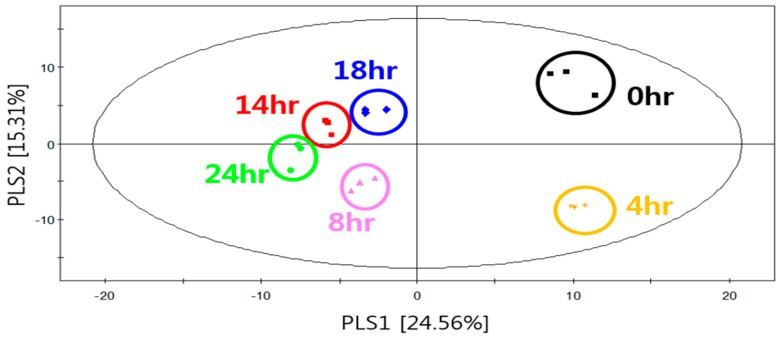
PLS-DA score plot for the volatile metabolites of of *S. fibuligera* KJJ81 in YPD medium according to the cultivation time.

Various microbial volatile metabolites have been detected during microbial growth. This study applied GC-MS equipped with SBSE to analyze the volatile metabolites of *S. fibuligera* KJJ81 in YPD medium according to the cultivation time. Figure 3 shows that PLS1 (24.56%) and PLS2 (15.31%) together explained 39.87% of the total variance. The parameters of the cross-validation modeling for the fourth PLS component were R2X = 0.587, R2Y = 0.782, and Q2Y = 0.416. Permutation testing (100 times) yielded R2 = 0.407 and Q2 = –0.498. Classifying the plots according to the cultivation time revealed that the profiles of the volatile metabolites moved from right to left along the PLS1 dimension with increasing cultivation time (Figure 3). The extracts of *S. fibuligera* KJJ81 samples could be differentiated primarily according to their PLS, with the initial cultivation stage (at 0 and 4 h) being positioned on the positive axis, and the middle and later cultivation stages (at 8, 14, 18 and 24 h) being positioned on the negative axis.

The major volatile metabolites (based on a criterion of VIP > 1.0) determined from *S. fibuligera* KJJ81 cultivated in YPD medium, including two acids, seven alcohols, two aldehydes, three benzene and benzene derivatives, seven esters, three furan and furan derivatives, one hydrocarbon, two lactones and five pyrazines (Table 2). Esters and alcohols comprised the largest group of volatile metabolites. Most esters produced by *S. fibuligera* KJJ81 in YPD media increased up to 14 h and then subsequently decreased. 3-Methylbutyl acetate, which has banana-like odor characteristics in wine, was quantitatively the predominant ester detected in *S. fibuligera* KJJ81. Xu et al. reported that these esters are formed by the esterification of alcohols with acids, or the biosynthesis by alcohol acetyltransferase using higher alcohols and acetyl-CoA as substrates [31]. 2-Phenylethanol was the dominant major volatile metabolite in the extracts cultivated in YPD medium, and its content increased continuously with the cultivation time. 2-Phenylethanol is one of the fusel alcohols and is formed from phenylalanine via the Ehrlich pathway following transamination, decarboxylation, and dehydrogenation, and is commonly detected as a major volatile of Korean rice wines such as *makgeolli*, providing a floral and rose-like aroma note [7]. The major volatile metabolites contributing fruity and flowery notes to the aroma of *makgeolli* and wine (3-methyl-1-butanol, 2-phenylethanol, 3-methylbutyl acetate, and 2-phenylethyl acetate) were formed from *S. fibuligera* KJJ81 cultivated in YPD medium.

### 2.3. Cultivation-time-dependent Nonvolatile Metabolites of S. fibuligera KJJ81 in YPD Medium

**Figure 4:**
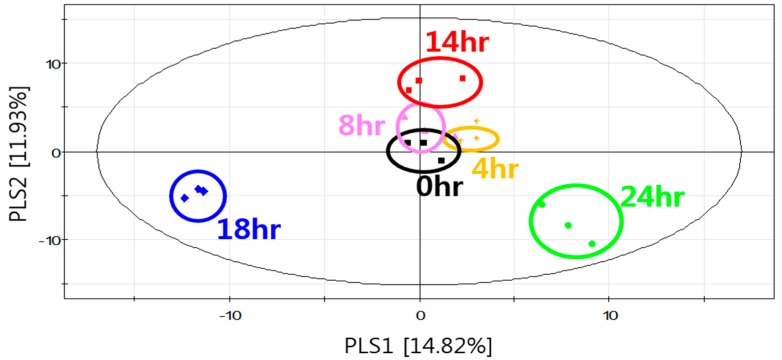
PLS-DA score plot for the nonvolatile metabolites of of *S. fibuligera* KJJ81 in YPD medium according to the cultivation time.

Nonvolatile metabolites in the biomass of *S. fibuligera* KJJ81 cultivated in YPD medium were analyzed using GC coupled to time-of-flight mass spectrometry (GC–TOF/MS) after derivatization. *S. fibuligera* KJJ81 samples cultivated for different times were clustered and distinguished using PLS-DA score plot (Figure 4). The PLS-DA score plot indicated that PLS 1 and PLS 2 explained 14.82% and 11.93% of the total variance, respectively. The cross-validation parameters for the fourth PLS component were R^2^X = 0.44, R^2^Y = 0.775 and Q^2^Y = 0.404. The R^2^ and Q^2^ values were 0.53 and –0.362, respectively, after permutation testing (100 times). Classifying the plots according to the cultivation time revealed that the nonvolatile metabolites separated into three groups on the PLS 1 dimension (Figure 4). PLS-DA was also applied to select major nonvolatile metabolites (based on a criterion of VIP > 0.7) related to the discrimination of samples according to the cultivation time. The following 17 major nonvolatile metabolites were identified in the biomass samples: three carbohydrates (mannose, arabitol, and mannitol), four lipids (propionic acid, palmitic acid, stearic acid, and myristic acid), two organic acids (oxalic acid and succinic acid), and eight amino acids (isoleucine, serine, alanine, glutamic acid, glycine, proline, phenylalanine, and threonine) (Table 3).

Mannose, arabitol, and mannitol were identified as major carbohydrates of *S. fibuligera* KJJ81 in YPD medium. Arabitol is mainly produced from glucose and other carbon sources containing sucrose and glycerol [32]. The level of arabitol increased up to 4 h and then subsequently decreased, whereas that of mannose decreased up to 8 h and then increased. Mannitol, which is a six-carbon sugar alcohol, can be found in various fungi and plants [33]. Onishi and Suzuki investigated the production of mannitol from glucose by osmophilic yeasts [34], and found that the level of mannitol remained constant during the initial cultivation stage and then increased markedly after 24 h of cultivation. This tendency could be explained by the synthesis of mannitol from glucose in YPD medium, since carbohydrates are consumed as carbon sources to provide microbial energy for growth of microorganisms via carbohydrate metabolic pathways [35].

Relatively high levels of palmitic acid and stearic acid were detected in *S. fibuligera* KJJ81 cultivated in YPD medium, and they changed continuously during the cultivation process. Palmitic acid, 16-carbon fatty acid, is a predominant fatty acid in eukaryotes [36]. In addition, Xiao, Dai, Zhu, and Yu reported that fatty acids are important for the flavor of Chinese rice wine, and that they originate mainly from raw materials [37]. Oxalic acid and succinic acid were detected as major organic acids in *S. fibuligera* KJJ81 cultivated in YPD medium, with oxalic acid predominating and its level being highest in *S. fibuligera* KJJ81 cultivated for 14 h. Oxalic acid is a simple dicarboxylic acid that is present in most living organisms [38].

The present study detected amino acids such as isoleucine, serine, alanine, glutamic acid, glycine, proline, phenylalanine, and threonine as major nonvolatile metabolites. The levels of most of the amino acids decreased up to 18 h of cultivation, which could be explained by their degradation via a metabolic pathway [39]. Amino acids could be degraded due to their utilization as nitrogen sources during early growth stage to maintain the nitrogen balance [40]. In addition, amino acids could be converted into esters, aldehydes, alcohols, sulfur-containing compounds, and lactones via the microbial metabolic pathway [39]. However, the levels of most amino acids increased at 24 h, which could be explained by their biosynthesis and also their degradation from proteins and peptides. These amino acids can be synthesized from keto acids via the tricarboxylic acid cycle in wine yeast [40,41]. Branched-chain amino acids (isoleucine) and aromatic amino acids (phenylalanine and tryptophan) convert into higher alcohols in yeast via the Ehrlich pathway [6], which involves transaminase, decarboxylase, and alcohol dehydrogenase mechanisms. The transamination of amino acids following decarboxylation and alcohol dehydrogenase reactions can generate α-keto acid, aldehyde, and alcohols, respectively [39]. Some of the enzymes associated with the transamination reaction are strongly expressed during the exponential phase and repressed during the stationary phase [41]. In the present study, the levels of certain amino acids such as isoleucine and phenylalanine, remained constant during exponential phase and then increased rapidly after stationary phase (18 h). The increase in amino acids after 18 h of cultivation in YPD medium could be explained by the need for *S. fibuligera* to effectively obtain access to external carbon and nitrogen sources thorough the degradation of proteins and peptides. On the other hands, the content of 2-phenylethanol, which is formed from phenylalanine via the Ehrlich pathway depend on the nitrogen sources, increased continuously with the cultivation time, in a similar pattern with 3-methyl-1-butanol. These results indicate that some volatile metabolites formed by *S. fibuligera* KJJ81 could be affected by nutrients such as carbohydrates and amino acids present in cultivation media.

In analyzing the genome of *S. fibuligera* KJJ81, numerous genes for extracellular hydrolytic enzymes, such as amylase, β-glucosidase, cellulase, and acidic protease, involved in saccharification and proteolysis, were discovered [42]. The observed expansion of hydrolytic enzymes in the *S. fibuligera* genome indicated that *S. fibuligera* was highly saccharolytic and proteolytic, resulting in its dominant appearance as a major yeast species in Asian traditional alcoholic starters. In addition, it was reported that *S. fibuligera* genes are expected to be applicable to the production of novel valuable enzymes and their metabolites [42].

## 3. Materials and Methods

### 3.1. Cultivation of S. fibuligera

*S. fibuligera* KJJ81 isolated from *nuruk* was cultivated in YP broth with different carbon sources such as glucose, fructose, galactose, xylose, and maltose. YP broth contains 1% (*w*/*v*) yeast extract, 2% peptone and 2% carbon source (glucose, fructose, galactose, xylose and maltose, 2% of each, respectively). *S. fibuligera* KJJ81 (initial OD_600_ = 0.1) was used for inoculation. Erlenmeyer flasks containing 40 mL of each media with screw cap were placed in a shaking incubator (Vision Scientific Co., Ltd., Bucheon-si, Gyeonggi-do, Korea) at 220 rpm and 37 °C for 18 h. The volatile and nonvolatile metabolites of *S. fibuligera* KJJ81 in YP medium containing glucose (YPD medium) were investigated according to the cultivation time. YPD medium contains 1% (*w*/*v*) yeast extract, 2% peptone and 2% glucose. *S. fibuligera* KJJ81 (initial OD_600_ = 0.1) were cultivated under the same conditions for 0, 4, 8, 14, 18, and 24 h.

### 3.2. Extraction and Analysis of Volatile Metabolites by GC-MS

Stir bar sorptive extraction (SBSE) was employed for the extraction of volatile metabolites in *S. fibuligera* KJJ81 samples cultivated in growth media. Samples were centrifuged at 4 °C and 3500 rpm for 300 s. Then, 8 mL supernatant was stirred at 1000 rpm for 60 min using polydimethyl siloxane coated stir bar (PDMS twister 10 mm length, 0.5 mm film thickness) (GERSTEL GmbH & Co., Mülheim an der Ruhr, Germany) to adsorb volatile metabolites. The stir bar was then washed with HPLC grade water (J.T. Baker, Avantor Materials, Pittsburg PA, USA) and dehydrated with lint-free tissue paper. The stir bar was inserted into twister desorption liner tubes (GERSTEL GmbH & Co.) and placed in a thermal desorption unit (TDU) (GERSTEL GmbH & Co.). The adsorbed volatile metabolites were thermally desorbed by increasing the temperature of TDU from 40 °C (0.5 min) to 280 °C (5 min) at a rate of 120 °C/min. Cooled injection system (CIS) was kept at −80 °C (0.01 min) and increased to 290 °C (1 min) at a rate of 12 °C/s Tenax TA™ (GERSTEL GmbH & Co.) was used as CIS liner. During desorption, CIS-4PTV temperature was maintained at −80 °C. Volatile metabolites were analyzed using a 7890B gas chromatograph (GC) system connected to a 5977A mass spectrometer (MS) (Agilent Technologies, Santa Clara, CA, USA). A DB-Wax column (30 m length × 0.25 mm i.d. × 0.25 μm film thickness, J&B Scientific, Folsom, CA, USA) was equipped and helium, a carrier gas, constantly flowed at 0.8 mL/min. Transfer line temperature was 300 °C and injection was performed in the splitless mode. GC oven temperature was held at 40 °C (5 min), followed by ramping to 230 °C (10 min) at 4 °C/min. The mass spectral data were obtained at 70 eV in electron ionization (EI), with a mass scan range of 35–350 amu and a scan rate of 4.5 scans/s.

### 3.3. Extraction and Analysis of Nonvolatile Metabolites by GC-TOF/MS

The extraction of nonvolatile metabolites of *S. fibuligera* KJJ81 was performed by fast filtration method, following the previous method [43]. One milliliter of sample was filtered through nylon membrane filter (LK LAB Korea Inc., Namyangju-si, Gyeonggi-do, Korea) under a vacuum. The filtered sample was washed with 5 mL of water and then vortexed with 20 mL of acetonitrile/water solvent (1:1, *w*/*v*) at −24 °C for 1 min. The mixture was put in liquid nitrogen for 15 min and thawed on ice. After completely thawing, the mixture was centrifuged at 3500 rpm and 4 °C for 20 min. Then 1 mL supernatant added with internal standards [20 μL threitol (0.1 mg/mL in water), 10 μL heptadecanoic acid (0.1 mg/mL in ethanol), 10 μL tropic acid (0.1 mg/mL in water) and 10 μL norleucine (0.1 mg/mL in water)] was vacuum-dried. Acetonitrile/water mixture (500 μL, 0 °C) (1:1, *w*/*v*) was transferred into the dried residue to eliminate lipids or wax of the extracts before further vacuum-dried. The vacuum-dried extracts were derivatized with 60 μL of methoxyamine hydrochloride (20 mg/mL in pyridine) for 60 min at 30 °C. Then, 80 μL of *N*,*O*-bis (trimethylsilyl)-trifluoroacetamide (BSTFA) with 1% trimethylchlorosilane (TMCS) was added to the samples and kept at 70 °C for 60 min. All solvents for derivatization agents used were purchased from Sigma-Aldrich (St. Louis, MO, USA). An Agilent 6890N GC coupled with time-of-flight (TOF) Pegasus III mass spectrometer (Leco, St. Joseph, MI, USA) was used to analyze non-volatile metabolites. Nonvolatile metabolites were separated on DB-5MS column (30 m length × 0.25 mm i.d × 0.25 μm film thickness, J&W Scientific). Flow rate of helium was 1 mL/min. Oven temperature was held at 80 °C (5 min), raised to 180 °C (5 min) at a rate of 10 °C/min, increased to 240 °C (5 min) at a rate of 8 °C/min, and then again to 290 °C (10 min) at a rate of 10 °C/min. The temperatures of injector and detector transfer line were 270 and 260 °C, respectively. Sample (1 μL) was injected in splitless mode. Mass scan range was 35 to 500 amu with a rate of 20 spectra/s. Ionization energy was 70 eV. All solvents and water used for extraction were HPLC grade and purchased from J.T. Baker (Philipsburg, NJ, USA).

### 3.4. Mass Spectral Data Processing of Volatile and Nonvolatile Metabolites

The data processing was employed using automated mass spectral deconvolution and identification system (AMDIS, http://chemdata.nist.gov/mass-spc/amdis/). Calibrating of retention index, baseline correction and deconvolution of mass spectrum were performed thorough AMDIS software. The parameters were as follows: component width set to 10, high resolution, low sensitivity and shape requirements set to high. The ELU files were created as output of AMDIS and the Spectconnect program (http://spectconnect.mit.edu) was performed to acquire a data matrix of retention times, normalized peak intensities, and accurate masses (*m*/*z*).

### 3.5. Identification and Quantification of Volatile and Nonvolatile Metabolites

The identification of volatile metabolites was carried out on the base of their mass spectra and retention index (RI) values. Comparison of the mass spectra was based on the NIST08 and Wiley 9 mass spectral libraries (Agilent Technologies, Palo Alto, CA, USA). The RI values of volatile metabolites were calculated with an alkane mixture from C_7_ to C_30_ as external standards. Volatile metabolites were quantified by comparing their peak areas to that of the internal standard, (1*R*,2*S*,4*R*)-1,7,7-trimethylbicyclo[2.2.1]heptan-2-ol((+)-borneol) (100 ppm *w*/*v* in methanol). The relative peak area (%) was an average of triplicate measurements.

Non-volatile metabolites were identified by comparing retention times and mass spectra with those of authentic reference compounds. GC-TOF/MS raw data were obtained from ChromaTOF™ software (Leco). All non-volatile metabolites were identified using Fiehn library, mainlibrary, Wiley 9 and in-house library. For quantification of non-volatile metabolites, 20 μL threitol (100 ppm *w*/*v* in water) for carbohydrates, 10 μL heptadecanoic acid (100 ppm *w*/*v* in hexane) for lipids, 10 μL tropic acid (100 ppm *w*/*v* in water) for organic acids and 10 μL norleucine (100 ppm *w*/*v* in water) for amino acids were used as internal standards.

### 3.6. Statistical Analysis

Partial least squares discriminant analysis (PLS-DA) was applied with SIMCA-P software (version 11.0, Umetrics, Umea, Sweden) to determine the metabolomic data variance among different *S. fibuligera* KJJ81 samples. The PLS-DA score plot indicated variances by PLS1 and PLS2. The differential variables were selected based on the variable importance plot (VIP) >1.0 and VIP > 0.7 values obtained using the PLS-DA. In order to estimate the significant differences between *S. fibuligera* KJJ81 samples during cultivation, multivariate analysis of variance (MANOVA) was performed using SPSS (version 12.0, Chicago, IL, USA). Duncan’s multiple comparison test was applied to identify statistically significant different metabolites (*p* < 0.05).

## 4. Conclusions

The present application of a nontargeted metabolomic approach to data sets of the profiles of volatile and nonvolatile metabolites revealed that *S. fibuligera* KJJ81 samples could be distinguished according to different cultivation media and times. In particular, certain volatile metabolites such as 2-phenylethanol, 2-phenylethyl acetate, and ethyl phenylacetate were predominantly formed in a glucose cultivation medium (YPD medium). Most of these volatile metabolites have characteristic fruity and flowery odor notes that might affect the quality and organoleptic properties of foods. The extracts of *S. fibuligera* KJJ81 samples cultivated for different times could be clearly differentiated on the PLS-DA plots, with the initial cultivation stage (at 0 and 4 h) being positioned on the positive axis, and the middle and later cultivation stages (at 8, 14, 18, and 24 h) being positioned on the negative axis. In addition, certain nonvolatile metabolites—carbohydrates (mannose, arabitol, and mannitol), fatty acids (palmitic acid and stearic acid), organic acids (oxalic acid and succinic acid), and amino acids (isoleucine, serine, alanine, glutamic acid, glycine, proline, phenylalanine, and threonine)—were the main contributors to distinguish *S. fibuligera* KJJ81 samples according to cultivation times. These findings indicate that volatile and nonvolatile metabolites of samples cultivated with *S. fibuligera* KJJ81—which are strongly related to aroma and taste—can change significantly during the cultivation process. These results can be used to improve the quality and organoleptic properties of foods fermented by *S. fibuligera* KJJ81.

## Figures and Tables

**Table 1:** The major volatile metabolites identified in *S. fibuligera* KJJ81 samples cultivated in media containing different carbohydrates.

No.	RI ^1^	Major Volatile Metabolites ^2^	VIP ^3^	ID ^4^
**Media Containing Xylose and Galactose**				
***Alcohols***				
98	1560	1-Octanol	0.80	A
***Benzenes & Benzene derivatives***				
96	1429	1,3-Bis(2-methyl-2-propanyl)benzene	1.11	B
***Esters***				
161	2261	(2*E*,6*E*)-3,7,11-Trimethyl-2,6,10-dodecatrien-1-yl acetate	0.79	B
131	1680	4-(2-Methyl-2-propanyl)cyclohexyl acetate	0.74	B
***Furans***				
108	2394	5-[(2*E*)-2-Octen-1-yl]dihydro-2(3*H*)-furanone	0.74	B
**Media Containing Glucose**				
***Alcohols***				
187	1910	2-Phenylethanol	1.42	A
175	1709	(2*E*)-3,7-Dimethyl-2,6-octadien-1-ol	1.25	B
***Aldehydes***				
181	1750	3-Dodecenal	1.42	B
199	1287	Octanal	1.42	B
172	1429	2-Octenal	1.33	B
***Esters***				
183	1813	2-Phenylethyl acetate	1.12	A
182	1782	Ethyl phenylacetate	0.90	A
163	<1100	Ethyl acetate	0.87	A
***Lactones***				
193	2424	6-Heptyltetrahydro-2*H*-pyran-2-one	1.43	A
**Media Containing Fructose and Maltose**				
***Alcohols***				
97	1452	1-Octen-3-ol	1.59	A
212	1659	2-Furylmethanol	1.51	A
106	2003	Phenol	1.25	A
91	1218	3-Methyl-1-butanol	0.86	A
***Esters***				
220	1128	3-Methylbutyl acetate	1.26	A
***Furans***				
83	2375	5-Hexyldihydro-2(3*H*)-furanone	0.87	A
***Sulfur-containing compounds***				
8	1059	(Methyldisulfanyl)methane	0.84	A

^1^ Retention indices were determined by using n-alkane mixture (containing C_8_–C_20_) as external references. ^2^ Major volatile metabolites were selected according to VIP values (with a criterion of VIP > 0.7). ^3^ All volatile metabolites listed by the order of their VIP values. ^4^ Identification: A, using authentic standards; B, having identical mass spectrum with library (Fiehn library, replibrary, mainlibrary and Wiley 9).

**Table 2:** Volatile metabolites identified in *S. fibuligera* KJJ81 according to cultivation time.

VIP ^1^	Volatile Metabolites ^2^	RI ^3^	Relative Peak Area (%) ^4^					
			0 h ^5^	4 h	8 h	14 h	18 h	24 h
***Acids***								
1.17	Butyric acid	1625	N.D. ^6^ a ^7^	0.02 ± 0.01 a	0.09 ± 0.04 ab	0.27 ± 0.02 d	0.14 ± 0.03 bc	0.24 ± 0.14 cd
1.36	2-Methylpropanoic acid	1568	N.D. a	N.D. a	0.01 ± 0.00 a	0.02 ± 0.00 b	N.D. a	0.04 ± 0.01 c
***Alcohols***								
1.10	2-Methyl-1-propanol	1113	N.D. a	0.04 ± 0.01 a	0.43 ± 0.06 b	0.59 ± 0.07 c	0.44 ± 0.01 b	0.46 ± 0.00 b
1.17	(2*E*)-3,7-Dimethyl-2,6-octadien-1-ol	1847	N.D. a	N.D. a	0.01 ± 0.01 a	0.04 ± 0.00 b	0.03 ± 0.00 b	0.03 ± 0.01 b
1.17	Phenol	1997	0.03 ± 0.01 bc	0.01 ± 0.00 a	N.D. a	0.03 ± 0.01 bc	0.02 ± 0.00 b	0.04 ± 0.01 c
1.19	1-Butanol	1160	2.22 ± 0.93 b	0.91 ± 0.04 a	0.32 ± 0.03 a	0.41 ± 0.06 a	0.25 ± 0.01 a	0.32 ± 0.02 a
1.27	2,4-Bis(2-methyl-2-propanyl)phenol	2307	0.18 ± 0.01 d	0.13 ± 0.01 ab	0.14 ± 0.01 bc	0.15 ± 0.01 bc	0.12 ± 0.00 a	0.16 ± 0.02 c
1.29	3-Methyl-1-butanol	1220	0.18 ± 0.05 a	2.24 ± 0.12 b	4.25 ± 0.21 c	5.02 ± 0.48 d	4.67 ± 0.26 cd	4.85 ± 0.28 d
1.40	2-Phenylethanol	1908	0.21 ± 0.04 a	2.88 ± 0.12 b	4.63 ± 0.35 c	5.40 ± 0.40 d	5.38 ± 0.32 d	6.18 ± 0.41 e
***Aldehydes***								
1.23	2-Furaldehyde	1458	0.04 ± 0.01 a	0.01 ± 0.00 a	0.02 ± 0.00 a	0.07 ± 0.02 bc	0.10 ± 0.05 c	0.05 ± 0.01 ab
1.47	Octanal	1287	N.D. a	0.01 ± 0.00 b	N.D. a	0.06 ± 0.01 d	0.07 ± 0.0037 c	0.03 ± 0.00 c
***Benzen and benzene derivatives***								
1.25	Benzaldehyde	1518	0.16 ± 0.05 b	0.04 ± 0.01 a	0.05 ± 0.05 a	0.03 ± 0.00 a	0.03 ± 0.00 a	0.03 ± 0.00 a
1.31	1-(2-Aminophenyl)ethanone	2210	0.01 ± 0.00 a	0.02 ± 0.00 b	0.02 ± 0.00 b	0.02 ± 0.00 ab	0.02 ± 0.00 a	0.02 ± 0.00 a
1.35	3-Methylbenzaldehyde	1642	0.05 ± 0.02 c	N.D. a	N.D. a	0.06 ± 0.01 cd	0.03 ± 0.00 b	0.07 ± 0.01 d
***Esters***								
1.11	Ethyl butyrate	<1100	N.D. a	N.D. a	0.44 ± 0.07 b	1.15 ± 0.13 d	0.91 ± 0.05 c	0.79 ± 0.08 c
1.14	(2*E*)-3,7-Dimethyl-2,6-octadien-1-yl acetate	1708	N.D. a	N.D. a	0.03 ± 0.00 c	0.03 ± 0.01 c	0.03 ± 0.00 c	0.02 ± 0.00 b
1.15	3-Methylbutyl acetate	1130	0.08 ± 0.01 a	0.30 ± 0.01 a	2.29 ± 0.16 c	4.14 ± 0.36 e	3.48 ± 0.17 d	1.59 ± 0.06 b
1.17	2-Phenylethyl acetate	1812	0.05 ± 0.01 a	0.25 ± 0.01 b	1.27 ± 0.12 c	1.63 ± 0.14 d	1.27 ± 0.08 c	1.56 ± 0.08 d
1.27	(2*E*,6*E*)-3,7,11-Trimethyl-2,6,10-dodecatrien-1-yl acetate	2260	0.02 ± 0.01 a	0.02 ± 0.00 a	0.09 ± 0.01 c	0.07 ± 0.02 b	0.09 ± 0.01 c	0.07 ± 0.00 b
1.37	Ethyl 3-phenylacrylate	2125	N.D. a	0.16 ± 0.01 b	0.35 ± 0.01 d	0.47 ± 0.05 e	0.22 ± 0.01 c	0.33 ± 0.04 d
1.45	Butyl 3-phenylacrylate	2333	N.D. a	0.07 ± 0.01 b	0.09 ± 0.01 b	0.09 ± 0.01 b	0.08 ± 0.01 b	0.07 ± 0.02 b
***Hydrocarbons***								
1.18	Hexadecane	1598	0.02 ± 0.00 ab	0.01 ± 0.00 a	0.01 ± 0.01 a	0.02 ± 0.00 bc	0.02 ± 0.01 c	0.02 ± 0.00 bc
***Lactones***								
1.21	6-Heptyltetrahydro-2*H*-pyran-2-one	2423	N.D. a	0.02 ± 0.00 b	0.06 ± 0.00 c	0.14 ± 0.02 e	0.09 ± 0.01 d	0.10 ± 0.01 d
1.22	6-Pentyltetrahydro-2*H*-pyran-2-one	2189	N.D. a	0.02 ± 0.00 b	0.05 ± 0.00 c	0.06 ± 0.01 c	0.06 ± 0.00 c	0.06 ± 0.00 c
1.25	5-Hexyldihydro-2(3*H*)-furanone	2139	0.05 ± 0.00 a	0.27 ± 0.03 b	0.66 ± 0.01 cd	0.74 ± 0.09 d	0.61 ± 0.02 c	0.65 ± 0.06 cd
1.27	5-[(2*Z*)-2-Octen-1-yl]dihydro-2(3*H*)-furanone	2393	N.D. a	0.03 ± 0.01 b	0.16 ± 0.01 c	0.23 ± 0.03 d	0.21 ± 0.01 d	0.17 ± 0.02 c
1.41	5-Pentyldihydro-2(3*H*)-furanone	2023	0.01 ± 0.00 a	0.07 ± 0.00 b	0.08 ± 0.02 bc	0.10 ± 0.02 c	0.08 ± 0.00 b	0.09 ± 0.01 bc
***Pyrazines***								
1.16	2-Isopropyl-5-methylpyrazine	1413	0.01 ± 0.00 a	0.02 ± 0.00 b	0.01 ± 0.01 a	0.01 ± 0.00 a	0.01 ± 0.00 a	0.01 ± 0.00 a
1.17	3-Ethyl-2,5-dimethylpyrazine	1446	0.03 ± 0.01 a	0.03 ± 0.00 a	0.04 ± 0.00 b	0.03 ± 0.01 a	0.03 ± 0.00 a	0.04 ± 0.00 b
1.21	2-Butyl-3,5-dimethylpyrazine	1610	0.02 ± 0.00 a	0.02 ± 0.00 a	0.02 ± 0.00 a	0.03 ± 0.00 c	0.03 ± 0.01 c	0.02 ± 0.00 b
1.22	2,5-Dimethylpyrazine	1320	0.08 ± 0.01 ab	0.11 ± 0.01 c	0.09 ± 0.01 b	0.07 ± 0.01 a	0.07 ± 0.01 a	0.07 ± 0.01 a
1.25	3-Butyl-2,5-dimethylpyrazine	1658	N.D. a	N.D. a	0.01 ± 0.00 a	0.10 ± 0.05 b	0.01 ± 0.00 a	0.01 ± 0.00 a

^1^ Variable importance in projection (VIP > 1.0). ^2^ All major volatile metabolites listed by the order of their VIP values. ^3^ Retention indices (RI) were determined by using n-alkane mixture (C_8_–C_20_) as external references. ^4^ Average of relative peak areas compared to that of the internal standard ± standard deviation (n = 3). ^5^ Cultivation times ^6^ Not detected. ^7^ Significant differences (*p* < 0.05) between *S. fibuligera* KJJ81 samples by using Duncan’s multiple comparison test.

**Table 3:** Nonvolatile metabolites identified in *S. fibuligera* KJJ81 according to cultivation time.

VIP ^1^	Non-Volatile Metabolite ^2^	MS ^3^	Relative Peak Area (%) ^4^					
			0 h ^5^	4 h	8 h	14 h	18 h	24 h
***Carbohydrates***								
1.46	Mannose	147	0.56 ± 0.03 d ^6^	0.11 ± 0.09 b	0.01 ± 0.00 a	0.02 ± 0.00 a	0.09 ± 0.01 b	0.48 ± 0.01 c
1.19	Arabitol	217	0.35 ± 0.31 b	1.07 ± 0.84 c	0.24 ± 0.02 a	0.04 ± 0.00 a	0.01 ± 0.00 a	0.28 ± 0.01 a
0.73	Mannitol	147	0.01 ± 0.00 a	0.01 ± 0.00 a	0.01 ± 0.01 a	0.01 ± 0.00 a	N.D. ^7^ a	0.03 ± 0.00 b
***Lipids***								
1.87	Propionic acid	147	0.22 ± 0.01 c	0.09 ± 0.01 b	0.23 ± 0.04 c	0.01 ± 0.00 a	0.50 ± 0.04 d	0.16 ± 0.01 b
1.37	Palmitic acid	117	3.90 ± 0.22 b	4.87 ± 0.16 c	3.75 ± 0.40 b	3.06 ± 0.34 a	3.74 ± 0.43 b	3.03 ± 0.37 a
1.30	Stearic acid	117	4.19 ± 0.23 b	5.26 ± 0.14 c	4.19 ± 0.38 b	3.38 ± 0.43 a	4.26 ± 0.50 b	3.31 ± 0.39 a
1.01	Myristic acid	117	0.12 ± 0.01 ab	0.18 ± 0.03 d	0.14 ± 0.01 bc	0.14 ± 0.01 bc	0.10 ± 0.01 a	0.11 ± 0.02 ab
***Organic acids***								
1.53	Oxalic acid1	147	0.01 ± 0.00 a	1.19 ± 2.02 b	1.76 ± 2.99 c	0.01 ± 0.01 a	N.D. a	0.01 ± 0.00 a
1.48	Oxalic acid2	147	9.95 ± 1.78 b	5.30 ± 1.70 a	5.12 ± 1.87 a	28.11 ± 2.14 d	10.22 ± 1.66 b	18.37 ± 1.69 c
0.92	Succinic acid	147	0.01 ± 0.01 a	0.01 ± 0.00 a	0.01 ± 0.01 a	0.02 ± 0.03 a	N.D. a	0.03 ± 0.06 a
***Amino acids***								
1.40	Isoleucine	158	0.01 ± 0.00 a	0.01 ± 0.001 a	0.01 ± 0.00 a	0.01 ± 0.00 a	0.03 ± 0.00 b	0.15 ± 0.00 c
1.38	Serine	204	0.01 ± 0.00 b	0.06 ± 0.00 e	0.01 ± 0.00 b	0.03 ± 0.00 c	0.00 ± 0.00 a	0.05 ± 0.00 d
1.20	Alanine	116	0.27 ± 0.01 c	0.40 ± 0.03 d	0.17 ± 0.07 b	0.30 ± 0.03 c	0.01 ± 0.00 a	0.26 ± 0.02 c
1.03	Glutamic acid	246	0.01 ± 0.00 a	0.10 ± 0.01 b	0.01 ± 0.00 a	0.01 ± 0.00 a	0.01 ± 0.00 a	0.01 ± 0.01 a
0.96	Glycine	102	0.05 ± 0.01 b	0.15 ± 0.03 c	0.02 ± 0.02 ab	0.04 ± 0.01 b	N.D. a	0.05 ± 0.01 b
0.96	Proline	142	0.20 ± 0.00 c	0.04 ± 0.03 ab	0.08 ± 0.07 b	0.24 ± 0.01 c	N.D. a	0.25 ± 0.01 c
0.85	Phenylalanine	218	0.01 ± 0.00 a	0.01 ± 0.00 a	0.01 ± 0.00 a	0.01 ± 0.00 a	0.01 ± 0.00 a	0.11 ± 0.01 b
0.81	Threonine	117	0.01 ± 0.00 b	0.03 ± 0.00 c	0.01 ± 0.00 ab	0.01 ± 0.00 a	0.01 ± 0.00 a	0.03 ± 0.00 b

^1^ Variable importance in projection (VIP > 0.7). ^2^ All major non-volatile metabolites listed by the order of their VIP values. ^3^ Quant mass of compounds. ^4^ Average of relative peak areas compared to that of the internal standard ± standard deviation (n = 3). ^5^ Cultivation times. ^6^ Significant differences (*p* < 0.05) between *S. fibuligera* KJJ81 samples by using Duncan’s multiple comparison test. ^7^ Not detected.
